# Germline 3p22.1 microdeletion encompassing *RPSA* gene is an ultra-rare cause of isolated asplenia

**DOI:** 10.1186/s13039-021-00571-0

**Published:** 2021-11-15

**Authors:** Aleksandra Oszer, Katarzyna Bąbol-Pokora, Sylwia Kołtan, Agata Pastorczak, Wojciech Młynarski

**Affiliations:** 1 Department of Pediatrics, Oncology and Hematology, Medical University of Lodz, Lodz, Poland; 2 Department of Pediatric Hematology and Oncology, Collegium Medicum, Nicolaus Copernicus University Torun, Bydgoszcz, Poland

**Keywords:** Isolated congenital asplenia, ICA, RPSA, Deletion

## Abstract

**Background:**

Isolated Congenital Asplenia (ICA, OMIM #271400) is a rare, life-threatening abnormality causing immunodeficiency, which is characterized by the absence of a spleen. Diagnosis should be completed in early childhood and antibiotic prophylaxis applied with additional vaccinations.

**Case presentation:**

We report the case of a six-month old girl with hematologic abnormalities and asplenia documented in imaging, with Howell-Jolly bodies in peripheral blood smear. Targeted Next Generation Sequencing screening did not reveal any pathogenic variant in genes associated with congenital asplenia. Since absence of the spleen was found by imaging, high-resolution copy number variations detection was also performed using genomic Single Nucleotide Polymorphism microarray: a heterozygous 337.2 kb deletion encompassing the *RPSA* gene was observed, together with *SLC25A38*, *SNORA6*, *SNORA62* and *MOBP* genes. Despite haploinsufficiency of *SLC25A38*, *SNORA6*, *SNORA62* and *MOBP*, no change in the clinical picture was observed. A search of available CNV databases found that a deletion of the *RPSA* locus seems to be unique and only duplications were found in this region with the frequency of less than 0.02%.

**Conclusions:**

Copy number variations in *RPSA* gene locus are ultrarare cause of isolated asplenia. Furthermore, since the patient does not present any concomitant clinical features, it would appear that haploinsufficiency of *SLC25A38*, *SNORA6*, *SNORA62* and *MOBP* genes does not affect the phenotype of patients. However, to confirm this thesis a longer follow-up of the patient’s development is needed.

**Supplementary Information:**

The online version contains supplementary material available at 10.1186/s13039-021-00571-0.

## Background

Isolated Congenital Asplenia (ICA, OMIM #271400) is a type of ribosomopathy characterized by the absence or hypoplasia of the spleen at birth [1]. Despite this, patients with ICA lack other developmental anomalies, and present as healthy individuals at birth. ICA occurs with a frequency of at least 0.51 per one million newborns per year [2]; as it is characterized by primary immunodeficiency [3], increasing the risk of severe presentation of bacterial infections, especially concerning *Streptococcus pneumoniae*, *Haemophilus influenzae* and *Neisseria meningitidis* [4] it can be a life-threating condition: patients with ICA have more than a 30-fold greater risk of invasive pneumococcal disease than the general population [5]. The rapid initiation of empiric antibiotic therapy in patients presenting with a fever corresponds with significantly-reduced mortality in patients with asplenia [6]. Moreover, antimicrobial prophylaxis and immunization with vaccines has the potential to eliminate approximately 80% of invasive infections [7]. Therefore, early diagnosis is crucial for ensuring a positive clinical outcome among patients with ICA.

Regarding the genetic background of the disease, at least three genes, viz*. GJA1*, *HMOX1* and *RPSA*, have been described as defective in isolated congenital asplenia [1, 8]. Moreover, a few multi-organ genetic defects have been associated with asplenia, including Stormorken syndrome (OMIM#185070) [6, 9], an autosomal recessive disorder called autoimmune polyendocrinopathy-candidiasis-ectodermal dystrophy (APECED, OMIM#240300) [10] or X-linked visceral heterotaxy type-1 (OMIM#306955) [11, 12]. Thus, an early genetic evaluation is necessary for a final clinical diagnosis.

We would like to present the diagnostic journey for a child with asplenia and haploinsufficiency of the *RPSA* gene coexisting with *SLC25A38*, *SNORA6*, *SNORA62* and *MOBP* gene deletion.

## Case presentation

Our patient was female, almost six months old, the first child of unrelated, healthy Caucasian parents. The perinatal history was complicated by gestational diabetes. The girl was delivered on term via spontaneous vaginal labor at 36 weeks of pregnancy due to a premature rupture of membranes. Her birth weight was 2340 g and she received 9 points on the Apgar scale. There were no abnormalities in the healing of the umbilicus. The girl was rehabilitated from the beginning of the third month of life on account of increased muscle tone (hypertonia). Furthermore, she had an episode of acute diarrhea at three months of age, apart from which she did not suffer from any other illness. She was fully vaccinated by 6 months of age.

The patient presented with episodic diarrhea, intermittent “sun-setting eye phenomenon” and mild facial dysmorphism to a rural hospital where ultrasonography revealed hepatomegaly with numerous diffuse areas and decreased echogenicity, as well as failure to visualize the spleen twice. An echocardiogram disclosed atrial septal defect (ASD) type 2 and treatment with spironolactone was introduced. The structures of the brain were correctly visualized in a transcranial ultrasound. Moreover, family history of lupus erythematosus was only positive in the mother’s sister.

The patient was transferred to the hematology department due to high white blood cell count (WBC), increased hemoglobin and platelet count, with no abnormalities in the peripheral blood smear, accompanied by a suspicion of congenital asplenia. Due to the good clinical condition of the patient, but with a not high persistent WBC (up to 20 K/μl) and Hb (up to 17 g/dl), further scheduled diagnostics were planned. In addition, due to the suspicion of immunity disorders related to congenital asplenia, antibiotic prophylaxis (amoxicillin) and additional vaccinations against *Streptococcus pneumoniae* and *Neisseria meningitidis* were ordered, as well as a postponement of “live vaccinations”. Cocoon vaccination was also ordered for the patient's family against influenza with an inactivated vaccine.

**Fig. 1:**
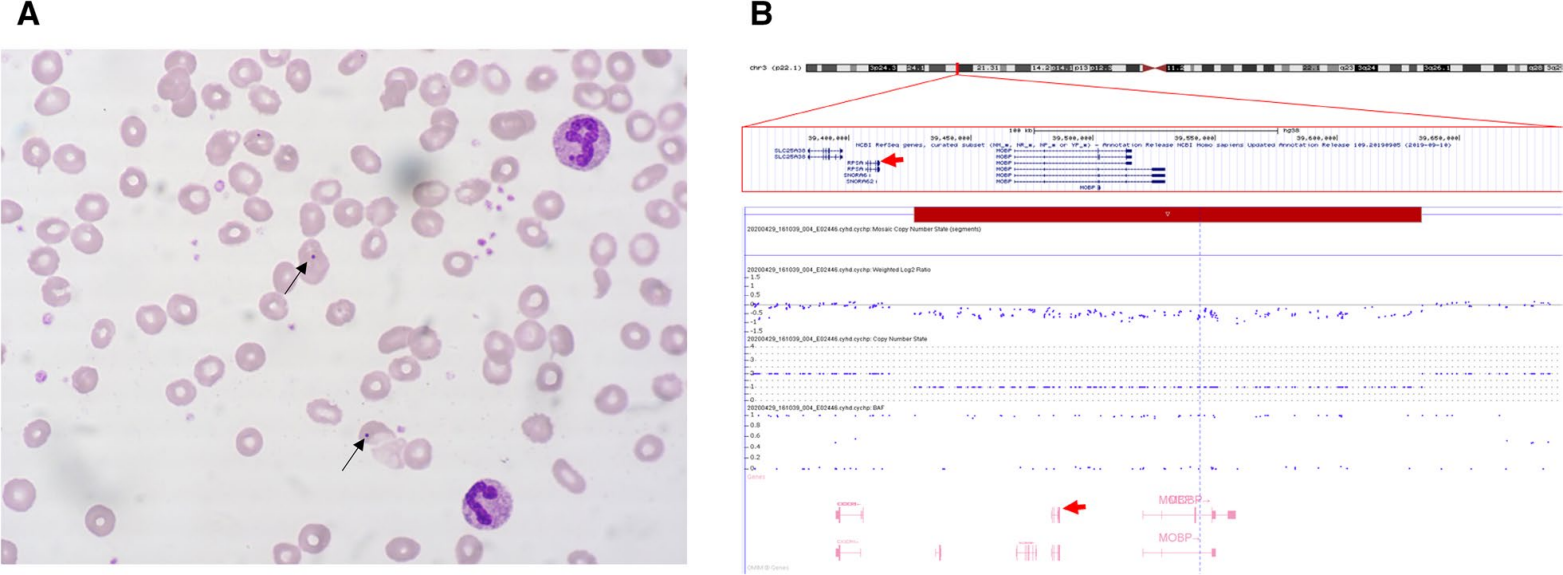
Laboratory and genetic findings in the patient with isolated asplenia. **A** Peripheral blood smear with Howell–Jolly bodies (indicated with arrows); **B** Heterozygous deletion at the short arm of chromosome 3 with a size of 337.2 kbp (arr[GRCh37] 3p22.2p22.1(39357060_39694267) × 1) encompassing the *RPSA* gene (red arrow)

During further diagnostics and subsequent hospitalizations, bone marrow trephine biopsy was performed, which showed no hematopoietic abnormalities. Laboratory tests revealed an increase in WBC (up to 41 K/μl) with the normal platelet and red blood cell count, slightly increased activity of aspartate aminotransferase (AST) and Alpha Fetoprotein (AFP) concentration within the age range. Subsequently, a spontaneous normalization of WBC was observed. The peripheral blood smear did not raise oncological concern but revealed Howell-Jolly bodies characteristic of asplenia (Fig. 1A). The following ultrasonography confirmed the absence of a spleen. Angio-CT revealed a developed, although rather narrow, splenic artery, directed to the left epigastric region, and the absence of a normal splenic vein. Consulting medical geneticist did not confirm any dysmorphic features and performed cytogenetic evaluation revealed constitutional karyotype as normal 46, XX. The absence of any cardiovascular abnormalities (except ASD) helped exclude heterotaxy syndromes such as Ivemark syndrome associated with asplenia [13]. Subsequent observations at age of one 12 and 16 months revealed resolution of any additional symptoms, which allows us to contribute to the diagnosis of ICA. Due to the high risk of ICA-related immune disorders, a full panel exam of immunoglobulin classes, subclasses and lymphocyte subpopulations was performed. No aberrations were found in the major immunoglobulin classes and IgG1-4 subclasses nor in the percentage distribution of the primary lymphocyte subpopulations. However, absolute values were elevated due to lymphocytosis. In a detailed analysis of the B lymphocyte subpopulation, the percentage of non-switched memory B cells slightly decreased while cells with the CD27-IgD phenotype increased.

Subsequent observations revealed resolution of symptoms such as disturbances in muscle tension, after rehabilitation. A thorough assessment, including neurological consultation, did not release any defects, and the development was evaluated as adequate to her age. Further clinical and laboratory observations did not reveal any abnormalities therefore, we suspect that haploinsufficiency of *SLC25A38*, *SNORA6*, *SNORA62* and *MOBP* genes does not affect the phenotype of patient.

### Genetic testing

Targeted Next Generation Sequencing for a panel of genes associated with primary immunodeficiencies and hematological defects was applied [1, 8, 9] (complete list of genes in Additional file 1: Table S1). This included genes associated with asplenia i.e. *GJA1*, *HMOX1*, *RPSA*, *STIM1* and *AIRE* [1, 8, 9]. Based on this approach no genetic defect was identified. However, only within a locus of *RPSA* gene homozygosity of the known SNP were noticed (Additional file 2: Fig. S1A), which might be suggestive for deletion in respective interval. Thus, this implied to perform a confirmatory searching for copy number variation. A dense genomic SNP microarray approach revealed approximately 50% of signal reduction in 312 SNPs located at *RPSA* locus which corresponds to a 337.2 kb deletion covering a fragment at the short arm of chromosome 3 (arr[GRCh37] 3p22.2p22.1(39357060_39694267) × 1), affecting five genes, including the *RPSA* gene (OMIM #150370) which encodes the SA ribosomal protein (Fig. 1B). This was the only CNV identified in this patient. None of the parents was a carrier of the same deletion (Additional file 2: Fig S1B and C). This confirms the diagnosis of Congenital Isolated Asplenia (ICAS, OMIM # 271400) resulting from de novo occurring monoallelic deletion of the *RPSA* gene.

Search for copy number variations at 3p22 using the International Standards for Cytogenomic Arrays databases using Decipher version 9.32 revealed that among 40,448 CNV records only 9 affected *RPSA,* including eight duplications (CNV frequency less than 0.02%). No deletion was found. This suggests that CNVs at the 3p22 are very rare phenomena occurring in germline human genome, and our patient could be treated as unique.

Literature review on haploinsufficiency in incidentally founding *SLC25A38*, *SNORA6*, SNORA62 and *MOBP* genes (Table 1) revealed possible abnormalities, which, however, did not occur in the described patient.

**Table 1:** List of the genes located within in a deleted interval at the short arm of chromosome 3

Deleted gene	Mode of inheritance (OMIM #)	Linked disease	Gene product function	Clinical characteristics in presented case	Reference
SLC25A38	AR (205950)	Autosomal Congenital Sideroblastic Anemia (anemia sideroblastic 2, pyridoxine-refractory)	Mitochondrial glycine transporter that imports glycine into the mitochondrial matrix. The encoded protein is required during erythropoiesis and is important for the biosynthesis of heme	–	Guernsey et al. [14]
RPSA	AD (271400)	Isolated Congenital Asplenia (ICA)	Ribosomal protein SA, a component of the small subunit of the ribosome, that could support controlling the production of certain proteins, which may be important in development before birth. It is also a cell surface receptor, specific for laminin	Absence of the spleen	Bolze et al. [1]
SNORA6	AD	Distal Arthrogryposis Type 6 (Arthrogryposis and sensorineural deafness)	–	–	https://www.genecards.org/
SNORA62	NA	Not known	–	–	–
MOBP	Not known	Amyotrophic Lateral Sclerosis	MOBP (Myelin Associated Oligodendrocyte Basic Protein) could be responsible for compacting or stabilizing the myelin sheath, likely by binding the negatively-charged acidic phospholipids of the cytoplasmic membrane	–	van Rheenen et al. [15]

## Material and methods

Next Generation Sequencing was performed using a custom-designed SureSelect QXT panel (Agilent Technologies, Santa Clara, USA) comprising 652 genes (Additional file 1: Table S1), related to primary immunodeficiencies and hematological diseases, including asplenia. The DNA library was prepared according to the manufacturer’s protocols and sequenced on a Next Seq 550(Illumina, San Diego, USA) during a 300 bp paired-end run. The data analyses of the target regions were performed using BWA Genome Alignment Software and the GATK Variant Caller algorithms and mapped to the human genome reference sequence GRCh37/hg19. The results were next analyzed using Variant Studio v. 3.0 (Illumina, USA) and Integrative Genomics Viewer v.2.3. Sequence analysis initially focused on the *GJA1, HMOX1, RPSA, STIM1* and *AIRE* genes, whose defects may lead to congenital asplenia. The filtering criteria included coverage with at least 20 reads, which was achieved for 98.6% of target regions, and minor allele frequency (MAF) below 0.01 in GnomAD database. All filtered variants were investigated by several bioinformatics tools: SIFT, Mutation Taster, and PolyPhen-2. The pathogenicity of the revealed changes was estimated in accordance with the American College of Medical Genetics and Genomics (ACMG) and based on ClinVar and HGMD, database. However, screening of *GJA1, HMOX1, RPSA, STIM1* and *AIRE* genes did not reveal any pathogenic or likely pathogenic variant.

Due to the patient presenting with a clinical disease and the eventuality of occurrence of Copy Number Variations, which cannot be determined by the targeted NGS, chromosomal microarray analysis was performed.

Being aware of the limitations of NGS through the Chromosomal microarray analysis (CMA) study, we then looked for deletions in genes related to congenital asplenia. SNP microarray analysis was performed using the Affymetrix CytoScanHD array (Thermo Fisher Scientific, CA, USA) which contains 2.67 million probes, and provides an mean resolution of one marker per 3 Kb in the target and one marker per 5 Kb in the backbone. The hybridization and scanning was performed using the GeneChip Fluidics Station 450Dx system with the GeneChip Scanner 3000 (Thermo Fisher Scientific, CA, USA) according to the CytoScanHS 1.2 protocol. Data obtained were analyzed using Chromosome Analysis Software (ChaS) v. 4.0 (Thermo Fisher Scientific, CA, USA) with the filtering criteria as 100 kbp, and 50 probes for the Copy Number Variations (CNVs).

### Database search

The International Standards for Cytogenomic Arrays (ISCA) (http://dbsearch.clinicalgenome.org/search/) databases were searched for patients harboring constitutive copy number alterations (CNAs) within short arm of chromosome 3p22.1 using Decipher ver 9.32 (https://decipher.sanger.ac.uk/).

## Discussion and conclusions

The diagnosis of congenital genetic defects is extremely important, especially when the clinical picture is unclear in infants and newborns, in whom we may not see all the symptoms of these syndromes. It is highly advantageous to identify the mutations in the genes responsible for asplenia as this condition may coexist with other disorders, and the patients are at high risk of severe bacterial infections [3, 5, 7, 16].

Our present study describes a patient with isolated asplenia associated with a mutation in the *GJA1, HMOX1 or RPSA* gene [1, 8]. A few syndromes are associated with asplenia. For instance, Stormorken syndrome is a genetic disorder caused by defects in *STIM1*, characterized by asplenia with tubular aggregate myopathy, thrombocytopenia or platelet dysfunction, miosis, ichthyosis, short stature, dyslexia and migraines [6, 9]. A rare autosomal recessive condition also exists called APECED, characterized by defects in the *AIRE* gene, causing symptoms associated with autoimmunity, endocrinopathy, ectodermal dystrophy and susceptibility to *Candida* infections [10]. Last but not least, X-linked visceral heterotaxy type 1, caused by a mutation in the *ZIC3* gene, results in transposition of thoracic and/or abdominal organs; although most defects in this syndrome concern the heart, asplenia may also appear in the clinical picture. It is also interesting to note that the panel designed to detect mutations in genes responsible for asplenia did not include the *ZIC3* gene [11, 12].

Based on the literature, the first diagnostic choice to identify the genetic defect leading to asplenia was targeted next-generation sequencing; however, surprisingly, no mutations causing asplenia were identified. Various genetic databases, including Online Mendelian Inheritance in Man (OMIM) and Human Gene Mutation Database (HGMD), indicate that the majority of pathogenic variants in *RPSA*, *GJA1* or *STIM1* genes affect single nucleotides, which should be detected by NGS. However, due to evident clinical picture of the patient’s disease and the eventuality of occurrence of Copy Number Variations (CNVs), which cannot be determined by the targeted NGS, another genetic method was performed.

Thus, NGS was supplemented by screening for copy number variation to enable simultaneous detection of deletions from the gene loci represented on the microarrays, with the resolution limited only by the number and size of probes used for the construction of microarrays [17]. Some researchers suggest that CMA should be the gold standard in clinical diagnostics for patients suspected of congenital anomalies and developmental disabilities who have no pathological variants identified in NGS [18]. In the present case, SNP microarray analysis identified the genes causing asplenia in this region which NGS did not. Indeed, it has been recommended that an array technology for high resolution copy number variant detection should be used in parallel with NGS to identify single copy amplifications and hemizygous deletions which are not detectable by NGS [19].

Based on this two-step approach, we were able to identify a 337.2 kbp deletion encompassing the *RPSA* gene whose defects appear to be associated with isolated asplenia. This is the first report showing a deletion of the whole of *RPSA* as a cause of isolated asplenia. A search of available CNV databases found that a deletion of the *RPSA* locus seems to be unique and only duplications or UPDs were found in this region with the frequency of 0.02%. Similarly, only family with small germline deletion at 3p22.1 locus was described in literature. However, this disturbed *UKL4* gene located 1.8 Mbp apart from *RPSA* locus and the patients showed neurological features and obesity with no asplenia [20]. These data highly support our statement that the deletion encompassing *RPSA* locus causing asplenia is an exceptional observation. Moreover, since the patient does not present any concomitant clinical features, it would appear that haploinsufficiency of *SLC25A38*, *SNORA6*, *SNORA62* and *MOBP* genes does not affect the phenotype of patients. However, to confirm this thesis a longer follow-up of the patient’s development is needed.

## Supplementary Information

**Additional file 1: Table S1.** The panel used in the study, including genes whose defects lead to primary immunodeficiencies and hematological disorders.

**Additional file 2: Fig. S1.** Additional evidence for causative role of the de novo deletion 3p22.1. A—NGS results using IGV software showing homozygosity for all genetic variations found at locus. *RPSA* in patient with asplenia. B, C—SNP array results of the mother and father, respectively, showing no copy number variations at 3p22.1.

## Data Availability

All data generated or analyzed during this study are included in this published article (and its Additional files).

## Abbreviations

ICA: Isolated congenital asplenia
NGS: Next generation sequencing
SNP: Single nucleotide polymorphism
APECED: Autoimmune polyendocrinopathy-candidiasis-ectodermal dystrophy
WBC: White blood cell
ASD: Atrial septal defect
AST: Aspartate aminotransferase
AFP: Alpha Fetoprotein
MAF: Minor allele frequency
ACMG: American College of Medical Genetics and Genomics
CMA: Chromosomal microarray analysis
ChaS: Chromosome Analysis Software
CNVs: Copy number variations
ISCA: International Standards for Cytogenomic Arrays
CNAs: Copy number alterations
OMIM: Online Mendelian Inheritance in Man
HGMD: Human Gene Mutation Database
UPD: Uniparental disomy
