# Supplementary material for: Germline 3p22.1 microdeletion encompassing RPSA gene is an ultra-rare cause of isolated asplenia
Source: Mol Cytogenet. 2021 Nov 15;14:51. doi: 10.1186/s13039-021-00571-0 (PMC8591925; doi:10.1186/s13039-021-00571-0)
Supplement: Supplementary file 1 — Additional file 1: Table S1. The panel used in the study, including genes whose defects lead to primary immunodeficiencies and hematological disorders. [file 13039_2021_571_MOESM1_ESM.docx]

Supplementary table 1. The panel used in the study, including genes whose defects lead to primary immunodeficiencies and hematological disorders.

| *ABCC6* | *ABCB7* | *ABCG5* | *ABCG8* | *ACD* | *ACP5* | *ACTB* | *ACTN1* |
| --- | --- | --- | --- | --- | --- | --- | --- |
| *ACVRL1* | *ADA* | *ADAM17* | *ADAMTS13* | *ADAR* | *ADAR1* | *AICDA* | *AIRE* |
| *AK1* | *AK2* | *ALAS2* | *ALDOA* | *AMN* | *ANK1* | *ANKRD26* | *ANO6* |
| *AP1S3* | *AP3B1* | *AP3D1* | *APOL1* | *ARPC1B* | *ASXL1* | *ATG16L1* | *ATM* |
| *ATP6AP1* | *B2M* | *BACH2* | *BCL10* | *BCL11B* | *BLM* | *BLNK* | *BLK* |
| *BLOC1S3* | *BLOC1S6* | *BMPR2* | *BPGM* | *BRCA1* | *BRCA2* | *BRIP1* | *BTK* |
| *C15orf41* | *C1QA* | *C1QB* | *C1QC* | *C1R* | *C1S* | *C2* | *C3* |
| *C3AR1* | *C4A* | *C4B* | *C5* | *C6* | *C7* | *C8A* | *C8B* |
| *C8G* | *C9* | *CARD11* | *CARD14* | *CARD9* | *CARMIL2* | *CASP10* | *CASP8* |
| *CCBE1* | *CD19* | *CD226* | *CD247* | *CD27* | *CD36* | *CD3D* | *CD3E* |
| *CD3G* | *CD40* | *CD40LG* | *CD46* | *CD55* | *CD59* | *CD70* | *CD79A* |
| *CD79B* | *CD81* | *CD8A* | *CDAN1* | *CDC42* | *CDCA7* | *CEBPA* | *CEBPE* |
| *CECR1* | *CFB* | *CFD* | *CFH* | *CFHR1* | *CFHR3* | *CFHR5* | *CFI* |
| *CFP* | *CFTR* | *CHD7* | *CHST14* | *CIITA* | *CLCN7* | *CLPB* | *COPA* |
| *CORO1A* | *COX4I2* | *CR2* | *CRFB4* | *CSF2RA* | *CSF2RB* | *CSF3R* | *CST3* |
| *CTC1* | *CTLA4* | *CTPS1* | *CXCR2* | *CXCR4* | *CYBA* | *CYBB* | *CYCS* |
| *DBA2* | *DCLRE1B* | *DCLRE1C* | *DHFR* | *DKC1* | *DNAJC21* | *DNASE1L3* | *DNASE2* |
| *DNMT3B* | *DOCK2* | *DOCK8* | *DTNBP1* | *ELANE* | *ENG* | *ENO1* | *EPB41* |
| *EPB42* | *EPG5* | *EPO* | *ERAP2* | *ERCC4* | *ERCC6L2* | *ETV6* | *EXTL3* |
| *F10* | *F11* | *F12* | *F13A1* | *F13B* | *F2* | *F2R* | *F3* |
| *F5* | *F7* | *F8* | *F9* | *FAAP24* | *FADD* | *FANCA* | *FANCB* |
| *FANCC* | *FANCD2* | *FANCE* | *FANCF* | *FANCG* | *FANCI* | *FANCL* | *FANCM* |
| *FAS* | *FASLG* | *FAT4* | *FCGR2C* | *FCGR3A* | *FCN3* | *FERMT1* | *FERMT3* |
| *FGA* | *FGB* | *FGG* | *FLG* | *FLI1* | *FLNA* | *FOXC1* | *FOXC2* |
| *FOXN1* | *FOXP3* | *FPR1* | *FYB* | *G6PC3* | *G6PD* | *G6PT1* | *GATA1* |
| *GATA2* | *GATA3* | *GCLC* | *GDF2* | *GFI1* | *GFI1B* | *GGCX* | *GINS1* |
| *GLA* | *GLRX5* | *GNE* | *GP1BA* | *GP1BB* | *GP6* | *GP9* | *GPI* |
| *GPX1* | *GSR* | *GSS* | *GUCY2C* | *HAX1* | *HBA1* | *HBA2* | *HBB* |
| *HELLS* | *HK1* | *HMOX1* | *HOIL1* | *HOIP1 (* | *HOXA11* | *HPS1* | *HPS3* |
| *HPS4* | *HPS5* | *HPS6* | *HRG* | *HYOU1* | *ICOS* | *IFIH1* | *IFNAR2* |
| *IFNGR1* | *IFNGR2* | *IGHM* | *IGKC* | *IGLL1* | *NFKBIA* | *IKBKB* | *IKZF1* |
| *IL10* | *IL10RA* | *IL10RB* | *IL12B* | *IL12RB1* | *IL17F* | *IL17RA* | *IL17RC* |
| *IL18* | *IL18RAP* | *IL1RN* | *IL21* | *IL21R* | *IL23R* | *IL2RA* | *IL2RG* |
| *IL36RN* | *IL6* | *IL7R* | *INO80* | *IRAK1* | *IRAK4* | *IRF2BP2* | *IRF3* |
| *IRF5* | *IRF7* | *IRF8* | *IRGM* | *ISG15* | *ITCH* | *ITGA2B* | *ITGAM* |
| *ITGB2* | *ITGB3* | *ITK* | *ITM2B* | *JAGN1* | *JAK1* | *JAK3* | *KCNK3* |
| *KCNN4* | *KDM6A* | *KDSR* | *KLF1* | *KMT2A* | *KMT2D* | *KRAS* | *LAMTOR2* |
| *LAT* | *LCK* | *LIG1* | *LIG4* | *LMAN1* | *LPIN2* | *LRBA* | *LRRC8A* |
| *LYST* | *LYZ* | *MAD2L2* | *MAGT1* | *MALT1* | *MAP3K14* | *MASP2* | *MCFD2* |
| *MCM4* | *MCP* | *MECOM* | *MEFV* | *MKL1* | *MOGS* | *MPIG6B* | *MPL* |
| *MPO* | *MRE11* | *MS4A1* | *MSH6* | *MSN* | *MTHFD1* | *MVK* | *MYD88* |
| *MYH9* | *MYSM1* | *NBAS* | *NBEAL2* | *NBN* | *NCF1* | *NCF2* | *NCF4* |
| *NCSTN* | *IKBKG* | *NFAT5* | *NFKB1* | *NFKB2* | *NFKBIA* | *NHEJ1* | *NHP2* |
| *NLRC4* | *NLRP1* | *NLRP12* | *NLRP3* | *NOD2* | *NOP10* | *NOTCH3* | *NRAS* |
| *NSMCE3* | *NT5C3A* | *ORAI1* | *OSTM1* | *OTULIN* | *P2RY12* | *PALB2* | *PARN* |
| *PEPD* | *PGK1* | *PGM3* | *PHF9* | *PIEZO1* | *PIGA* | *PIK3CD GOF* | *PIK3R1* |
| *PKLR* | *PLA2G4A* | *PLAT* | *PLAU* | *PLCG2* | *PLEKHM1* | *PLG* | *PMS2* |
| *PNP* | *POLA1* | *POLE* | *POLE2* | *POMP* | *PRF1* | *PRKACG* | *PRKCD* |
| *PRKDC* | *PROC* | *PROS1* | *PSEN* | *PSENEN* | *PSMA3* | *PSMB4* | *PSMB8* |
| *PSMB9* | *PSTPIP1* | *PTEN* | *PTPN11* | *PTPN22* | *PTPRC* | *RAB27A* | *RAC2* |
| *RAD50* | *RAD51A* | *RAD51C* | *RAG1* | *RAG2* | *RANBP2* | *RASGRP1* | *RASGRP2* |
| *RBCK1* | *RBM8A* | *RELB* | *RFWD3* | *RFX5* | *RFXANK* | *RFXAP* | *RHAG* |
| *RHOH* | *RIPK1* | *RLTPR* | *RMRP* | *RNASEH2A* | *RNASEH2B* | *RNASEH2C* | *RNF168* |
| *RNU4ATAC* | *RORC* | *RPL11* | *RPL15* | *RPL26* | *RPL27* | *RPL35A* | *RPL5* |
| *RPS10* | *RPS17* | *RPS19* | *RPS24* | *RPS26* | *RPS27* | *RPS28* | *RPS29* |
| *RPS7* | *RPSA* | *RTEL1* | *RUNX1* | *SAMD9* | *SAMD9L* | *SAMHD1* | *SBDS* |
| *SEMA3E* | *SERPINC1* | *SERPIND1* | *SERPINE1* | *SERPINF2* | *SERPING1* | *SGPL1* | *SH2D1A* |
| *SH3BP2* | *SLC11A2* | *SLC19A2* | *SLC29A3* | *SLC35C1* | *SLC37A4* | *SLC39A4* | *SLC45A2* |
| *SLC46A1* | *SLC4A1* | *SLC7A7* | *SLFN14* | *SLX4* | *SMAD1* | *SMAD4* | *SMAD9* |
| *SMARCAL1* | *SMARCD2* | *SNX10* | *SOS1* | *SP110* | *SPINK5* | *SPTA1* | *SPTB* |
| *SRC* | *SRP54* | *SRP72* | *STAT1* | *STAT2* | *STAT3* | *STAT5B* | *STEAP3* |
| *STIM1* | *STK4* | *STN1* | *STX11* | *STXBP2* | *TAP1* | *TAP2* | *TAPBP* |
| *TAZ* | *TBK1* | *TBX1* | *TBXA2R* | *TBXAS1* | *TCF3* | *TCIRG1* | *TCN2* |
| *TERC* | *TERT* | *TFRC* | *TGfb1* | *THBD* | *THPO* | *TICAM1* | *TINF2* |
| *TIRAP* | *TLR3* | *TMC6* | *TMC8* | *TMEM173* | *TMPRSS6* | *TNFAIP3* | *TNFRSF11A* |
| *TNFRSF13B* | *TNFRSF13C* | *TNFRSF1A* | *TNFRSF4* | *TNFRSF6* | *TNFSF11* | *TNFSF12* | *TPI1* |
| *TPP1* | *TPP2* | *TRAC* | *TRAF3* | *TRAF3IP2* | *TREX1* | *TRNT1* | *TSR2* |
| *TTC37* | *TTC7A* | *TUBB1* | *TYK2* | *UBE2T* | *UNC119* | *UNC13D* | *UNC93B1* |
| *UNG* | *UROS* | *USB1* | *USP18* | *VIPAS39* | *VKORC1* | *VPS13B* | *VPS45* |
| *VWF* | *WAS* | *WDR1* | *WIPF1* | *WRAP53* | *XIAP* | *XRCC2* | *XRCC9* |
| *YARS2* | *ZAP70* | *ZBTB24* | *SEC23B* | *STAT4* | *ABCB1* | *ERCC2* | *ERCC3* |
| *CSTA* | *GJB3* | *GJB4* | *KRT1* | *KRT10* | *INAVA* | *HRAS* | *APOA1* |
| *APOA2* | *APOC2* | *APOC3* | *BRAF* | *CALR* | *CFHR2* | *CFHR4* | *COL17A1* |
| *COL1A1* | *COL7A1* | *CSF1R* | *CTSC* | *DGKE* | *DIAPH1* | *EPCAM* | *FUT2* |
| *GBA* | *GJA1* | *HABP2* | *HBD* | *HBG1* | *HBG2* | *HTRA2* | *ICOSLG* |
| *IL12RB2* | *IL17A* | *IL5* | *IL6R* | *IL7* | *IRF1* | *IRF4* | *ITGA2* |
| *ITGA3* | *KIF23* | *KLKB1* | *KNG1* | *LYN* | *MASTL* | *MRE11* | *MTR* |
| *MTRR* | *MYO5B* | *OFD1* | *PARN* | *PIGA* | *PKLR* | *PLEC* | *PUS1* |
| *RB1* | *RECQL4* | *RET* | *RPGR* | *RPL18* | *RPL35* | *RUNX3* | *SAMHD1* |
| *SCN1A* | *SERPINA10* | *SKIV2L* | *SLC25A38* | *STAT6* | *SUOX* | *TBX21* | *TF* |
| *TFPI* | *TGFB2* | *TGFB3* | *TGFBRII* | *TLR2* | *TLR5* | *TMEM50B* | *TNFSF15* |
| *TP53* | *TPM4* | *TRAF6* | *ZMIZ1* |  |  |  |  |
