# Supplementary material for: Germline 3p22.1 microdeletion encompassing RPSA gene is an ultra-rare cause of isolated asplenia
Source: Mol Cytogenet. 2021 Nov 15;14:51. doi: 10.1186/s13039-021-00571-0 (PMC8591925; doi:10.1186/s13039-021-00571-0)
Supplement: Supplementary file 2 — Additional file 2: Fig. S1. Additional evidence for causative role of the de novo deletion 3p22.1. A—NGS results using IGV software showing homozygosity for all genetic variations found at locus. RPSA in patient with asplenia. B, C—SNP array results of the mother and father, respectively, showing no copy number variations at 3p22.1. [file 13039_2021_571_MOESM2_ESM.pptx]

## Slide 1
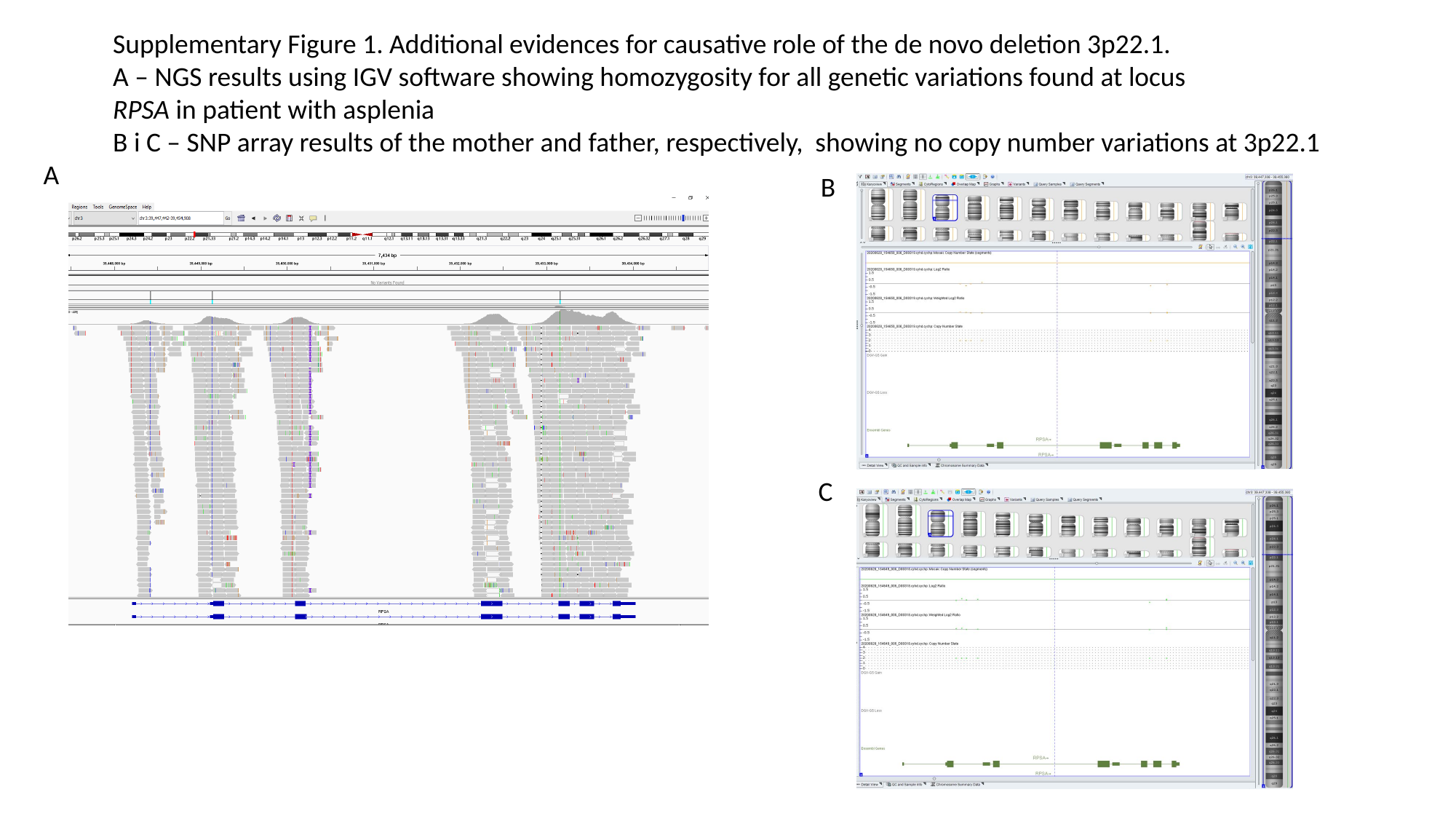

Supplementary Figure 1. Additional evidences for causative role of the de novo deletion 3p22.1.
A – NGS results using IGV software showing homozygosity for all genetic variations found at locus
RPSA in patient with asplenia
B i C – SNP array results of the mother and father, respectively, showing no copy number variations at 3p22.1
A
B
C
